# Understanding the Pro-Ana Subculture: Illness, Sickness or Choice

**DOI:** 10.1192/bjo.2024.227

**Published:** 2024-08-01

**Authors:** Iyinoluwa Popoola

**Affiliations:** King's College London, London, United Kingdom

## Abstract

**Aims:**

This digital ethnographic study explores the varying perceptions of anorexia within the pro-anorexia (pro-ana) subculture by utilizing Professor Marinker's framework of disease, illness, and sickness to qualitatively analyse how individuals within this community interpret anorexia as a personal experience, a societal role, or a deliberate choice.

**Methods:**

This study examines insights from the pro-ana community, obtained through pro-ana spaces on social media platforms and dedicated pro-ana online forums contributing to understanding their viewpoints on anorexia. Drawing from established literature on anorexia nervosa and the pro-ana subculture, this study employs a qualitative analysis of online pro-ana spaces, examining discussions, narratives, and beliefs shared within these communities. The study also integrates historical perspectives, cultural critiques, and psychological theories to offer a comprehensive understanding.

**Results:**

The pro-ana subculture presents diverse perspectives on anorexia, challenging traditional definitions of illness and sickness. Some individuals view anorexia positively, perceiving it as a means of discipline and self-improvement. Cultural and historical influences, including the feminine expectation, the normalization of the disordered eating habits of the ‘gym bro', and societal beauty standards, further shape perceptions within the pro-ana community. There is some debate on how the media influences the proliferation of eating disorders and the evolving definitions of anorexia – including the introduction of atypical anorexia. There is also an ascetic spirituality associated with anorexia, which can be seen as a matter of faith or delusion.

**Conclusion:**

The concept of anorexia within the pro-ana subculture challenges traditional definitions of illness, sickness, and choice. This study highlights the cultural, historical, and gendered dimensions influencing these perspectives. Understanding this complex interplay can inform mental health professionals, educators, and policymakers about the potential risks posed by pro-ana communities. It emphasizes the importance of preventive measures, media responsibility, and a nuanced approach to engaging with individuals influenced by the pro-ana subculture. Recognizing the multifaceted nature of anorexia within this community is crucial for developing effective interventions and support strategies for patients with anorexia who engage with the online pro-ana community.

